# Epicardial adipose tissue as a metabolically active visceral fat depot: an emerging target in cardiovascular disease

**DOI:** 10.3389/fcvm.2026.1824863

**Published:** 2026-07-28

**Authors:** Isabella Rojas-Bergés, Ricardo Mauricio Villamil-Galván, Santiago Salgado-López, Santiago Obregón-Rosas, Julio Granados-Montiel

**Affiliations:** 1 Facultad Mexicana de Medicina, Universidad La Salle México, Mexico City, Mexico; 2 Medical Education Department, Instituto Nacional de Cardiología Ignacio Chávez, Mexico City, Mexico; 3 Centre for Craniofacial & Regenerative Biology, Faculty of Dentistry, Oral & Craniofacial Sciences, King's College London, London, United Kingdom; 4 Instituto de Química, Unidad Mérida, Universidad Nacional Autónoma de México (UNAM), Mérida, Mexico

**Keywords:** adipokines, atrial fibrillation, cardiac fibrosis, cardiovascular disease, coronary artery disease, epicardial adipose tissue, metabolic syndrome, renin-angiotensin-aldosterone system

## Abstract

**Background:**

Epicardial adipose tissue (EAT) is a metabolically and endocrinologically active visceral fat depot with unique anatomical proximity to the myocardium, and is increasingly recognized as relevant to cardiovascular health and disease. Its anatomical position, in direct contact with the myocardium and coronary arteries without an intervening fascial barrier, is consistent with paracrine and vasocrine signaling toward adjacent cardiac structures.

**Objective:**

This review aims to provide a comprehensive analysis of EAT biology, from its physiological roles in lipid homeostasis, mechanical protection, and thermogenesis, to its pathological transformation in the context of obesity, metabolic syndrome, and coronary artery disease.

**Methods:**

A narrative review was conducted using PubMed/MEDLINE, Scopus, Web of Science, and Embase, focusing on studies published between January 2000 and December 2025. Human data—particularly from prospective cohorts, randomized trials, and meta-analyses—were prioritized over preclinical and *in vitro* work, with the latter invoked principally to support mechanistic discussion. Detailed search strategy and inclusion criteria are described in the Methods section.

**Results:**

Under pathological conditions, EAT undergoes structural and functional remodeling characterized by adipocyte hypertrophy, immune cell infiltration, dysregulated adipokine secretion, and a shift from a beige-like thermogenic phenotype to a white, pro-inflammatory depot. These changes promote cardiac fibrosis through activation of fibroblast-to-myofibroblast differentiation via TGF-*β*/SMAD and biomechanical pathways, contribute to arrhythmogenesis through disruption of connexin-mediated conduction and activation of the adipose-neural axis, and facilitate coronary atherosclerosis through local inflammatory signaling. The renin-angiotensin-aldosterone system plays a critical role in mediating EAT-related inflammation and fibrosis through AT1R-driven pro-inflammatory cascades, while the ACE2/Ang 1–7/MasR axis offers counter-regulatory cardioprotection.

**Conclusions:**

EAT is an emerging therapeutic target and candidate biomarker in cardiovascular medicine. Echocardiographic thickness and CT/MRI volumetric measurements have shown prognostic associations with adverse cardiovascular events, although standardized cut-off values across imaging modalities and large prospective outcomes trials specifically validating EAT-based risk stratification remain lacking. Strategies that may favorably modulate EAT remodeling, including GLP-1 receptor agonists and conceptual approaches to promote re-browning, represent emerging—but still preliminary—therapeutic directions whose independent cardiovascular benefit requires further investigation.

## 1 Introduction

Over the past few years, the multiple functions of epicardial adipose tissue (EAT), as well as its associations with cardiac pathology, have been increasingly recognized. EAT is now understood as a metabolically and endocrinologically active visceral fat depot that contributes to thermogenesis, provides mechanical protection to the heart, and may participate in repair and regeneration processes (1, 2). Increased EAT has been associated with coronary artery disease, atrial fibrillation, and heart failure with preserved ejection fraction (3). The mechanisms by which systemic inflammation may influence EAT and by which EAT, in turn, may contribute to systemic blood pressure regulation are still being delineated. Systemic inflammation—driven by obesity, insulin resistance, or chronic inflammatory states—is thought to alter EAT volume, secretory profile, and resident immune cell composition through circulating cytokines such as TNF-α, IL-6, and IL-1β, with macrophage polarization shifting toward a pro-inflammatory M1 phenotype within the depot. Conversely, EAT may contribute to systemic blood pressure regulation through several proposed mechanisms, including locally produced angiotensinogen and angiotensin II that may enter the systemic circulation, leptin-mediated activation of central sympathetic outflow, and reduced adiponectin output with downstream effects on endothelial nitric oxide bioavailability. It should be noted, however, that a recent meta-analysis confirmed a robust epidemiological association between EAT and hypertension while explicitly concluding that the causal link remains undetermined (4). The directionality of these relationships, and the relative contribution of EAT to systemic vs. purely local cardiac effects, therefore remains an area of active investigation and is treated as such throughout this review (5).

Studies of cardiac fat date back to the 19th century. At that time, pathologists like Richard Quain began their research on the adipose tissue deposited in the heart, due to a series of cases of sudden death with no apparent cause other than the presence of abnormalities in cardiac fat levels, as detailed in his work on fatty diseases of the heart. Quain described a difference between the fat surrounding the heart and the fat infiltrating the myocardium. Therefore, he postulated that the abnormal distribution of this fatty tissue could have pathophysiological implications (6). However, several authors had already published similar findings years earlier, giving rise to a new line of research that has gained interest in the 21st century. Among these authors, Bedford stands out, who documented, in a review of cases obtained since 1783, the excess fat accumulated and its relationship to the heart. Laennec, in his work on mediate auscultation, states that, according to the discoveries of Corvisart, a significant accumulation of fat around the heart could be a cause of sudden death (7, 8).

Currently, the study of EAT using ultrasound, magnetic resonance imaging (MRI), and computed tomography (CT) has been proposed. CT has been shown to allow the calculation of EAT volume, thus enabling its use as a biomarker for coronary artery disease (CAD) (9, 10). In ultrasound, the parasternal long-axis projection perpendicular to the right ventricle (RV) is used, allowing for the measurement of epicardial fat thickness. These measurements have linked epicardial fat thickness as a hemodynamic prognostic factor with adverse effects in heart failure with preserved ejection fraction (HFpEF). Therefore, it has been used as it is considered a safe, easily reproducible and non-invasive method that can be performed routinely (11).

In 2018, Morales-Portano and colleagues conducted a study in which they compared, using echocardiographic measurements, the thickness of epicardial adipose tissue and other risk factors in relation to their ability to predict adverse cardiovascular outcomes in patients with CAD. The results demonstrated that epicardial adipose tissue thickness was four times more predictive of major adverse cardiovascular events (MACEs) than BMI, left ventricular ejection fraction (LVEF), and coronary artery complexity in a study population with coronary artery disease (11). However, these results had already been obtained in previous studies, such as the 2014 prospective follow-up study by Nakanishi et al. of patients with coronary artery disease at risk of developing coronary plaques and future acute coronary syndrome. The prevalence of obstructive plaques and plaques with high-risk characteristics increased significantly in patients who showed an increase in EAT volume during follow-up. In contrast, no significant changes were observed in patients whose EAT volume remained constant or decreased (12).

Cardiac fibrosis is the excessive and persistent accumulation of extracellular matrix secreted by fibroblasts in response to chronic inflammation. Activated by TGF-*β* and angiotensin II, fibroblasts proliferate and differentiate into myofibroblasts via biomechanical and biochemical pathways, which together increase the gene expression of *α*-SMA and the synthesis of collagen I and III. Another fibrotic mechanism involves the renin-angiotensin-aldosterone system, particularly through the AT1R pathway, which has been associated with inflammation, oxidative stress, and profibrotic remodeling. It is important to note, however, that the pro-inflammatory and pro-fibrotic effects of RAAS activation may be at least partly dependent on the concomitant rise in blood pressure rather than direct receptor-mediated tissue effects: in the murine model of Kai et al. (13), hydralazine-mediated normalization of blood pressure substantially blocked Ang II–induced cardiac inflammation, monocytic fibroblast precursor infiltration, and myofibroblast formation. Counterevidence for blood-pressure-independent fibrotic mechanisms also exists—for example via the (pro)renin receptor (14)—and the relative contributions of pressure-dependent and pressure-independent pathways remain incompletely resolved. This distinction has direct interpretive consequences when considering EAT-derived RAAS components, as discussed in Section 4: locally produced angiotensin II within EAT may not necessarily reproduce the downstream cardiac effects observed when systemic activation is accompanied by hemodynamic change. Furthermore, dysfunctional epicardial adipose tissue, when associated with a profibrotic state, has been linked to altered connexin expression and slowed conduction, which may contribute to disruptions of the cardiac conduction circuit and to arrhythmias such as atrial fibrillation.

Epicardial adipose tissue has emerged as a key mediator linking systemic metabolic dysfunction to cardiovascular disease. In the context of metabolic syndrome, EAT undergoes profound structural and functional remodeling, shifting from a physiologically protective fat depot to a metabolically active and pro-inflammatory organ. Sharing embryological origin and biological behavior with visceral abdominal adipose tissue (VAT), EAT exhibits adipocyte hypertrophy, immune cell infiltration, and dysregulated adipokine secretion under conditions of insulin resistance and chronic low-grade inflammation. Due to its unique anatomical proximity to the myocardium and coronary arteries, without intervening fascial barriers, EAT is capable of exerting direct paracrine and vasocrine effects on adjacent cardiac structures (15).

## 2 Methods

This narrative review was conducted through structured searches of PubMed/MEDLINE, Scopus, Web of Science, and Embase, covering articles published between January 2000 and December 2025, with selected foundational references from earlier dates retained where historically pivotal. The principal MeSH terms and free-text keywords used included “epicardial adipose tissue,” “pericardial adipose tissue,” “perivascular adipose tissue,” “cardiac fibrosis,” “cardiac fibroblast,” “renin-angiotensin-aldosterone system,” “adipokines,” “atrial fibrillation,” “heart failure with preserved ejection fraction,” and combinations thereof using Boolean operators. Inclusion criteria comprised English-language original research, systematic reviews, and meta-analyses addressing EAT physiology, pathophysiology, imaging, biomarker performance, or therapeutic modulation. Editorials, letters without primary data, conference abstracts, and non-peer-reviewed preprints were excluded. Reference lists of included articles were screened for additional relevant work.

An explicit prioritization scheme was applied to the synthesis. Human data—particularly from prospective cohort studies, randomized controlled trials, and meta-analyses—were given primary weight in supporting clinical inference. Preclinical and *in vitro* evidence was invoked principally to support mechanistic discussion rather than clinical conclusions, and the species and experimental context of such evidence is explicitly noted at each major mechanistic discussion throughout the manuscript. Where conflicting findings exist in the literature, both sides have been presented. We acknowledge that this work is a narrative rather than systematic review; the methodological limitations attendant to that format, including the absence of formal risk-of-bias assessment and the potential for selection bias, are addressed in Section 9 (Limitations and Knowledge Gaps).

## 3 Epicardial adipose tissue in the healthy heart

The adult heart is composed of three distinct cell populations: the endocardial inner layer, the contractile myocardium, and the outer lining of the cardiac chambers, the epicardium. These layers are enveloped by the parietal pericardium, in its outermost portion, and the visceral pericardium in its innermost portion—the latter forming the epicardium (15–17).

Cardiac adipose tissue consists of fat deposits located between the myocardium and the pericardium. Due to their anatomical distribution, cardiac adipose tissue is divided into different subtypes. Clarifying the distinctions between these terms is essential for their understanding, primarily because their anatomical and embryological characteristics give them significantly different behaviors (18). Epicardial adipose tissue is located between the myocardium and the visceral layer of the pericardium. This compartment covers approximately 80% of the heart surface and represents about 20% of the total weight of the heart (19).

Corradi et al. studied the structural relation between the EAT and the myocardium, reporting a proportional relationship between epicardial fat and myocardial mass. Autopsy-based analysis demonstrated a greater presence of fat in the right ventricle compared with the left, establishing average epicardial fat/gram of cardiac muscle values of 0.15 g in men and 0.17 g in women for the left ventricle, and 0.48 g and 0.61 g, respectively, for the right ventricle. This structural relationship between the myocardium and the EAT appears to be preserved regardless of ischemia or hypertrophy. However, in the presence of a hypertrophic process, both adipose and myocardial tissue exhibit correlated growth in their masses (21). Nevertheless, given the metabolic and endocrine activity of EAT, it could be argued that the preservation of this structural relationship may conceal a degree of functional dissociation between these two compartments.

**Figure 1:**
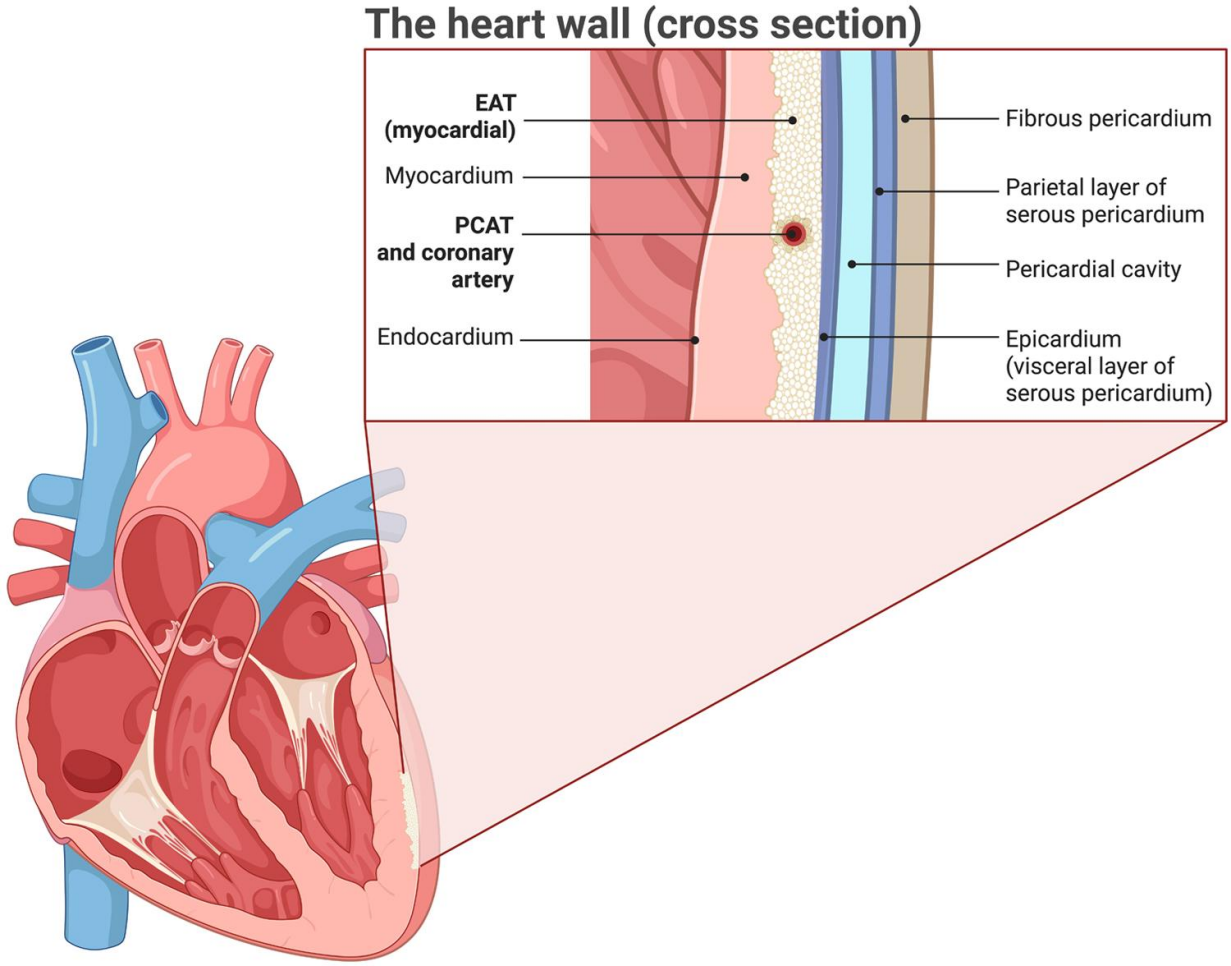
Anatomy of the heart wall from a cross section, based on (15–17, 19, 20, 22–25). Original work created at BioRender.com.

The EAT differentiates into pericoronary adipose tissue (PCAT), which surrounds the intima of the coronary arteries, and myocardial adipose tissue, located exclusively over the myocardium (21). The paracardial adipose tissue is located in the outer layer of the parietal pericardium, but within the mediastinum. Therefore, it has also been considered mediastinal fat. Lastly, the pericardial adipose tissue (PAT) consists of epicardial and paracardial adipose tissue (22, 23) (Figure 1).

Although PCAT has been considered a subdivision of EAT, it is also classified as perivascular adipose tissue (PVAT). PVAT is a highly dynamic and metabolically active tissue that surrounds various vascular beds, with the exception of the neural and pulmonary vasculature. It has also been shown to be a niche for stem and progenitor cells. These stromal cells give it the capacity to differentiate preadipocytes into white or brown adipose tissue. The composition of PVAT with respect to white or brown adipose tissue will depend on its location. In the case of PCAT, there is a greater amount of white adipose tissue. PVAT itself has a mechanical function by maintaining vascular homeostasis through the structural support of blood vessels. However, it has also demonstrated paracrine functions in the regulation of vascular remodeling, angiogenesis, and inflammation, and its dysregulation can lead to the development of endothelial and cardiovascular dysfunction (24, 25). Despite the anatomical relationship between the PCAT and EAT, their contribution to the physiology and pathology of cardiovascular diseases appears to differ. Consequently, these two adipose tissues have been studied separately, seeking to compare their clinical utility and relevance.

The EAT shares a common embryonic origin with intra-abdominal adipose tissue from splanchnopleuric mesoderm, and both originate from brown adipose tissue (26, 27). Anatomically, EAT is distributed in the atrioventricular and interventricular grooves, along the major branches of the coronary arteries, and it extends to the right ventricular surface, as well as to the apex of the left ventricle (28, 29).

Particularly, there is no connective tissue or fascia between the epicardial adipose tissue and the myocardium. This means that both share the same vascularization via the coronary arteries. Due to this specific characteristic of the EAT, it has been proposed that there is a direct paracrine interaction between the adipose tissue and the underlying myocardium (29, 30). This potential interaction highlights the importance of strictly maintaining the balance of the physiological functions of the EAT.

**Figure 2:**
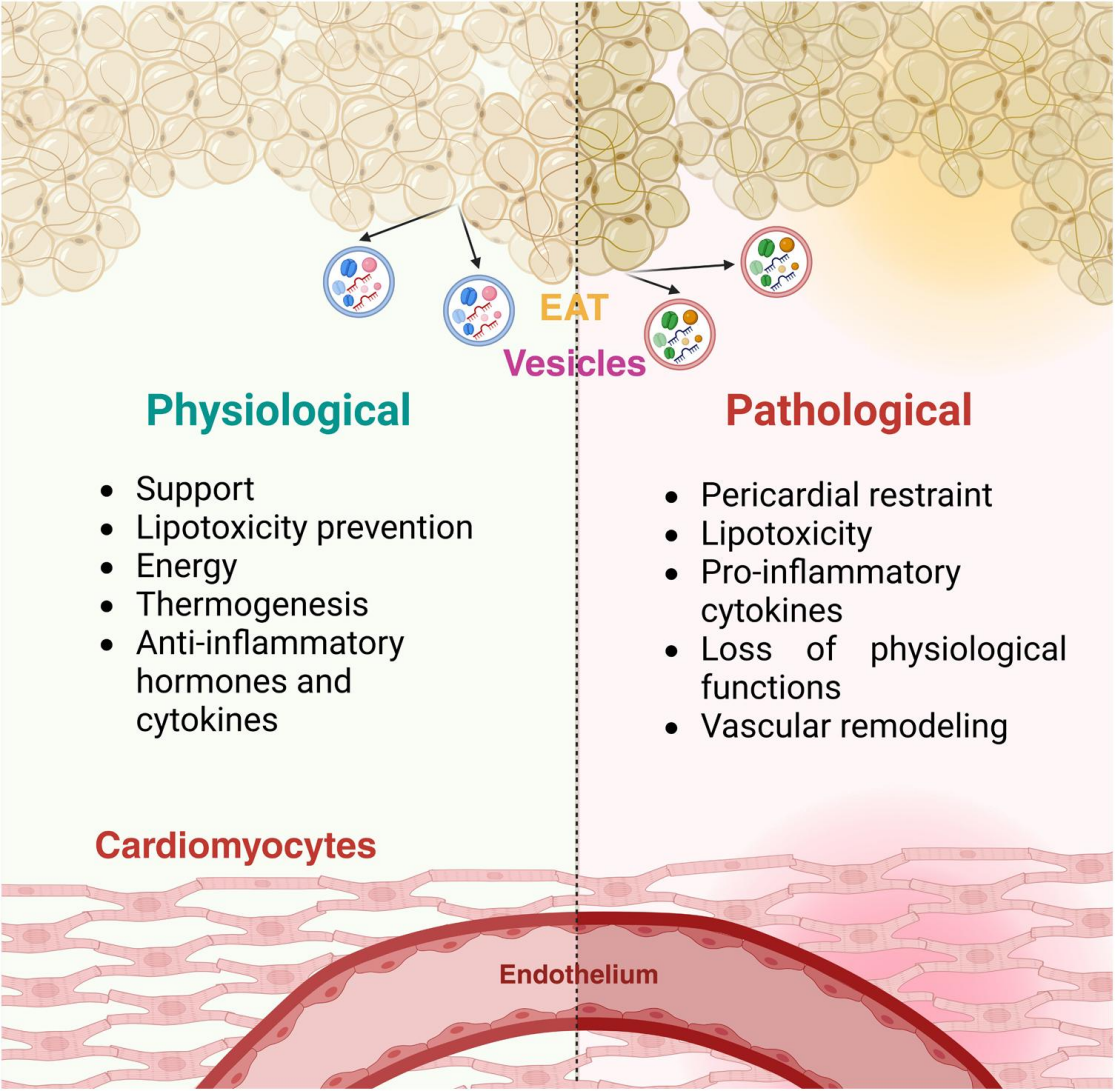
Comparison between physiological and pathological EAT characteristics, showing EAT, cardiomyocytes, endothelium and vesicles containing cytokines, miRNAs and hormones, based on (27, 31–39). Original work created at BioRender.com.

Epicardial adipose tissue is a complex physiological system that is not yet fully understood and whose functions are still under discussion. It is composed of multiple cell types, including inflammatory cells, immune cells, nerve cells, stromal cells, and vascular cells which have both cardioprotective and pathogenic properties (Figure 2). However, it is primarily composed of adipocytes (40). The characteristics of cardiac adipocytes have been studied in isolation, suggesting that epicardial adipose tissue possesses a multifunctionality that can be divided into mechanical, metabolic, and thermogenic functions (30).

### 3.1 Metabolic functions of EAT

EAT plays an important role in lipid homeostasis. Its anatomical location and lack of division with the myocardium allows direct communication with myocardial tissue mediated by cytokine secretion. On a nutritional level, it participates in the storage and mobilization of lipids. EAT releases free fatty acids, which serve as a major oxidative substrate for the myocardium. Because the myocardium is a tissue with a high metabolic demand, it is considered that EAT has a higher rate of release and uptake of free acids compared to other adipose tissue deposits, such as subcutaneous and visceral adipose tissue (30).

The myocardium takes up and metabolizes FFAs from the coronary arterial blood, and the oxidation of long-chain fatty acids accounts for approximately 70% of the ATP generated by the heart (31, 41). It should be emphasized that the heart does not produce energy *de novo*; rather, it converts the chemical energy stored in metabolic substrates into the ATP that powers mechanical contraction. Free fatty acids, especially medium- and short-chain fatty acids, function as an efficient substrate for mitochondrial beta-oxidation, which nourishes the myocardium bidirectionally via the coronary bloodstream or interstitial fluid. It has been suggested that this communication occurs between different cells, and from these stem the two theories about paracrine and vasocrine communication. The first proposes that adipokines secreted by epicardial adipocytes, stromal cells, and vascular cells travel through the interstitium and act primarily at the level of the coronary arteries. In contrast, the other suggests that the release of both adipokines and free fatty acids from epicardial tissue goes directly to the vasa vasorum, targeting the arterial wall via vasocrine communication (27, 32). More recent studies suggest a third theory, in which the EAT secretes extracellular vesicles containing cytokines and microRNAs, which could be responsible for the development of arrhythmias such as atrial fibrillation (AF) (33). Physiologically, the EAT acts as a buffer to absorb free fatty acids and prevent cardiotoxicity from high levels of these acids. The fatty-acid-binding protein 4 has been identified as highly expressed in the EAT, suggesting its association with fatty acid transport (34).

In addition, it would be appropriate to consider other little-studied perspectives on the characteristics of cardiac adipocytes. A study conducted by Litviňuková in 2020, which sought a deeper molecular understanding of heart cells, identified that cardiac adipocytes uniformly express glycerol-3-phosphate acyltransferase, mitochondrial (GPAM), fatty acid synthase (FASN), ADIPOQ, and LEP, at lower levels (36). GPAM is a gene that encodes a mitochondrial enzyme called GPAT1—one of the GPAT isoforms (36). GPAT1 is responsible for the first step in glycerolipid formation, catalyzing the conversion of glycerol-3-phosphate to lysophosphatidic acid (37). Its presence in cardiac adipose tissue suggests its potential as a metabolic regulator of myocardial energy demands. However, murine studies have shown that GPAT1 has immunological functions, associating its decrease or absence with a reduction in T lymphocyte proliferation and a significant decrease in interleukin-2 (IL-2) secretion following antigenic stimulation. In this context, dysregulation of this enzyme could contribute to myocardial inflammation in a paracrine manner (37).

On the other hand, FASN enzyme catalyzes the conversion of acetyl-CoA and malonyl-CoA into palmitate (42). Its identification in cardiac adipose tissue suggests its potential involvement in energy storage through the *de novo* synthesis of fatty acids, acting in conjunction with GPAT1. Although FASN research has focused on oncogenesis, its presence warrants further investigation to more precisely define its function in the heart and its potential contributions to cardiovascular pathology (38).

Interestingly, the presence of ADIPOQ and LEP has been previously studied as functional properties of EAT (32). However, understanding the EAT secretome is vital to integrating its role in the protective and harmful functions of this adipose tissue. The duality of these functions lies in the conditions of the microenvironment surrounding the EAT.

Mazurek et al. demonstrated in their study on human epicardial adipose tissue that, in patients with coronary artery disease, the EAT shows infiltration of inflammatory cells such as T lymphocytes (CD3), macrophages (CD68), and mast cells specifically in the microvasculature and connective tissue septa. In addition, it was reported the elevated local expression of MCP-1 and inflammatory cytokines (IL-1, IL-6, IL-6sR, and TNF-α), which did not correlate with plasma concentrations. These findings suggest that there may be an inflammatory microenvironment in the EAT that is not reflected at the level of systemic inflammatory markers, allowing the local progression of the inflammatory process despite apparently normal plasma cytokine concentrations (43).

This review explores some of the adipokines present in the EAT secretome, acknowledging that it is not exhaustive. This field is still under investigation, and due to the relatively short time since its initial study, some findings remain speculative. However, this review aims to present those adipokines that have proven useful for EAT function and whose dysregulation can lead to the development of cardiovascular diseases (Table 1). Nevertheless, it is worth emphasizing that a more in-depth study of all adipokines should be considered to standardize our understanding of the EAT secretome.

**Table 1:** Functional properties of epicardial adipose tissue bioactive molecules, based on (27, 31).

**Properties**	**Factors**
Pro-inflammatory, proatherogenic	TNF-α, MCP-1, IL1, IL1*β*, IL-1Ra, IL6, IL8, IL10, CRP, PAI-1, Prostaglandin D(2), haptoglobin, α1-glycoprotein, JNK, sPLA2-IIA, fatty-acid-binding protein 4, RANTES, ICAM
Growth Factors	NGF, FLT1
Anti-inflammatory, anti-atherogenic	Adiponectin, Adrenomedullin
Vascular remodeling, blood pressure control, myocardial hypertrophy, adipogenesis	Angiotensin, Angiotensinogen, Leptin
Insulin-mimetic, markers of visceral fat	Resistin, Visfatin, Omentin
Brown fat differentiation transcription factors	PRDM-16, PGC-1α
Thermogenic	UCP-1
Receptors	Angiotensin II type 1 receptor, TLRs, PPAR-*γ*, GLUT-4

CRP, C-reactive protein; FLT-1, soluble vascular endothelial growth factor receptor; GLUT-4, glucose transporter-4; ICAM, soluble intercellular adhesion molecule; IL, interleukin; IL-1Ra, interleukin-1 receptor antagonist; JNK, c-Jun N-terminal kinase; MCP-1, monocyte chemoattractant protein-1; NGF, nerve growth factor; PAI-1, plasminogen activator inhibitor-1; PGC-1α, PPAR-*γ* coactivator-1α; sPLA2-IIA, secretory type II phospholipase A2; PPAR-*γ*, peroxisome-proliferator-activated receptor *γ*; PRDM-16, brown adipocyte differentiation transcription factor PR-domain-missing16; RANTES, regulated upon activation normal T cell and secreted; TLRs, toll-like receptors; TNF-α, tumor necrosis factor-alpha; UCP-1, uncoupling protein-1.

#### 3.1.1 Adiponectin

Adiponectin is a 30 kDa protein derived from adipose tissue to which various functions have been attributed, including antiatherogenic, insulin-sensitizing, lipid oxidation-enhancing, and vasodilatory activities (43, 44). The effects of adiponectin are mediated by its receptors. Adipocytes are known to express the ADIPOR1 and R2 receptors, enabling autocrine and paracrine action of adiponectin in this tissue. Its anti-inflammatory effects are mediated by the suppression of the release of certain pro-inflammatory cytokines—such as interleukin-6 (IL-6) and tumor necrosis factor-alpha (TNF-α)—from adipocytes and surrounding vascular stromal cells (45). In an injured artery, adiponectin accumulates in the subendothelial space and reduces the expression of adhesion molecules of endothelial cells in response to an inflammatory stimulus (46). In parallel, adiponectin is structurally similar to C1q and is able to activate the classical complement pathway by binding to C1q through the globular domain of the A chain of C1q (47, 48).

Adiponectin is encoded by the ADIPOQ gene located on chromosome 3q27, which is considered a susceptibility locus for metabolic syndrome (MetS), type 2 diabetes, and cardiovascular disease (CVD) (49). Similarly, overexpression of ADIPOQ has been associated with decreased macrophage infiltration in adipose tissue (45). These effects may be due to the fact that circulating adiponectin concentration is associated with increased PPAR*γ* expression in adipose tissue. The role of PPAR*γ* in the regulation of inflammation has been studied, suggesting that its ligands are responsible for the suppression of TNF-α and IL-6 through the inhibition of NF-*κ*B. This translates into diseases with a pro-inflammatory state, such as obesity and insulin resistance, which are associated with decreased circulating adiponectin and elevated serum levels of IL-6 and TNF-α (50–52).

Iacobellis et al. were the first to report the expression of adiponectin in epicardial adipose tissue and its contribution to myocardial fatty acid combustion. In that same study, they found that patients with CAD had a decrease in adiponectin in the EAT of approximately 40%, suggesting that patients with CAD had less adiponectin than those without it (53). On the other hand, van Andel et al. address adiponectin as an acute-phase reactant, describing that during a myocardial infarction, adiponectin levels are elevated in both plasma and myocardial tissue. However, after the acute phase, adiponectin levels decrease. Therefore, decreased levels of adiponectin have been associated with chronic heart disease such as compensated heart failure (47).

Another property of adiponectin is its role in regulating myocardial redox status. The presence of Nox 1, 2, 4, and 5 in cardiovascular tissue contributes to both normal vascular and cardiac function, as well as to the development of cardiovascular diseases (54). Antonopoulos et al., in their study on adipose tissue and myocardial redox status, reported a positive association between O₂⁻ produced by NADPH and circulating adiponectin levels produced by the EAT, as well as ADIPOQ gene expression. This suggests a possible paracrine protective response by the EAT against myocardial oxidative stress through the inhibition of NADPH oxidase (55).

#### 3.1.2 Leptin

Leptin is a pleiotropic hormone that regulates many physiological processes, including food intake, body mass, reproductive function, inflammatory responses, fetal growth, angiogenesis, and lipolysis (55). Unlike the protective effects attributed to adiponectin, leptin has been associated with pro-inflammatory states and cardiovascular risk (56–58). These effects could be explained by the increase in the production of TNF and IL-6 by monocytes through the activation of JAK2–STAT3 (59).

Strict regulation of circulating leptin levels is necessary for proper cardiovascular function (a level of 5–15 ng/mL in lean individuals), leading to the postulation that this hormone functions in a paradoxical and dichotomous manner within the cardiovascular system. Low leptin levels promote cardiovascular disorders, as leptin signaling physiologically favors the balance of glucose metabolism and fatty acid oxidation in the heart. It also has cardioprotective effects against ischemia/reperfusion injury (60). Conversely, its absence leads to a lack of metabolic flexibility (61). Similarly, hyperleptinemia—commonly caused by obesity—is a trigger for cardiovascular dysfunction due to leptin resistance (62, 63).

Chen et al., in their rodent study on myocardial damage caused by leptin secreted from the EAT, demonstrated that in rodents with MetS, EAT-derived leptin levels were directly related to the level of myocardial damage. In rats with visceral obesity and EAT accumulation, adipokine secretion was dysregulated, with elevated leptin levels—independent of serum levels. The main mechanisms of myocardial damage caused by leptin were associated with oxidative stress and mitochondrial dysfunction via the PKC/NADPH oxidase/ROS pathway (64).

### 3.2 Mechanical functions of EAT

The coronary arteries are delimited by epicardial adipose tissue regardless of the body's habitus (64). Previous studies have suggested that one of the possible mechanical functions of the EAT is to buffer the coronary arteries against the torsion induced by the arterial pulse wave and cardiac contraction (65). Another proposal is its function as a buffer against physical stress generated by repeated cardiac movements, being an additional layer that protects the cardiac tissue (66, 67).

### 3.3 Thermogenic functions of EAT

Human EAT displays brown fat features and properties, as it expresses uncoupling protein 1 (UCP1), a brown adipocyte-specific mitochondrial protein, which executes thermogenesis via uncoupling of ATP synthesis from fat oxidation during cold (68). However, it has been observed that UCP-1 expression in the EAT decreases with age, thus losing its thermogenic properties. Its main functions include protecting the myocardium and coronary arteries against hypothermia, ischemia, or hypoxia (69).

Sacks et al. mention that histologically, epicardial adipose tissue shares certain characteristics with brown and white adipose tissue, and therefore should be considered beige adipose tissue (70). It is worth noting that beige adipocytes have a different cell lineage than brown adipose tissue, although they share certain characteristics and functions. Brown adipocytes originate from precursor cells that express myogenic factor 5 (Myf5). In contrast, white adipocytes derive from Myf5-negative precursors (71). It has been observed that beige adipocytes emerge from a precursor similar to that of white adipose tissue. This translates into lower UCP-1 expression compared to brown adipocytes, which require stimuli such as cold or other inducers such as β2-adrenergic agonists or PPARγ activators (72).

Therefore, the term “browning” has been proposed, in which white adipose cells induce a thermogenic function via UCP-1. These changes occur through different types of stimuli, which are still under investigation. However, Doukbi et al. report that the main stimuli include environmental factors such as cold exposure, physical exercise, and pharmacological treatment [specifically treatment with *β*3-adrenergic agonists and glucagon-like peptide-1 receptor (GLP-1R) analogs]. Furthermore, it is mentioned that interleukin-mediated intercellular communication also plays an important role in the browning of white adipose tissue, specifically IL-4, IL-5, IL-13, IL-17A, and IL-33 (73, 74).

## 4 EAT in the pathogenesis of heart disease

### 4.1 Fibrosis

Fibrosis is the excessive accumulation of fibrous connective tissue (comprising the extracellular matrix with its collagen and fibronectin components) at a site of inflammation or tissue damage in response to the inflammatory process. Physiologically, the deposition of fibrous connective tissue as part of the tissue repair process secondary to inflammation is essential, but when this process is repetitive or dysregulated, excessive deposition occurs persistently around the affected site. In the long term, this leads to organ dysfunction and permanent scarring, as the affected organ becomes trapped in a dense layer of fibrous connective tissue (75, 76).

### 4.2 Fibroblasts

The main components of the extracellular matrix are type I and III collagen fibers, which are synthesized by fibroblasts. These cells represent 10%–20% of the total cell population in cardiac tissue. Fibroblasts are highly heterogeneous cells, not specific to any one organ, but rather cell populations characterized by their ability to synthesize collagen and reside in connective tissue. In particular, cardiac fibroblasts (CFs) originate embryologically from the paraxial mesoderm of the proepicardial organ and can migrate to two zones, undergoing two types of differentiation. The most common type is migration to the epicardium, where they undergo an epithelial-to-mesenchymal transition to generate epicardial-derived fibroblast progenitors (EDFPs). The second type is of endothelial origin, as is the case with resident fibroblasts of the interventricular septum and right ventricle, which derive from valve endothelial cells via endothelial-to-mesenchymal transition (ETMT). Physiologically, fibroblasts in their quiescent state function to provide structure to cardiac tissue and maintain homeostasis of the heart's electrical conduction pathways. Cardiac fibroblasts are characterized by expressing the cellular markers CD90, DDR2, Sca1, FSP1, and vimentin, as well as synthesizing extracellular matrix components such as fibronectin, laminin, and collagens I and III (75–78).

### 4.3 Genetic role of cardiac fibroblasts in fibrosis

The population of CFs is determined by the expression of epicardial genes such as TCF21, WT1, and TBX18, with gene expression varying depending on the developmental stage. Embryonic fibroblasts are characterized by expressing only TBX20, a transcriptional modulator that regulates and promotes cardiac tissue proliferation, and whose expression decreases after birth. TCF21, also known as epicardin, is a class II protein of the basic helix-loop-helix type—a cell lineage-specific dimeric transcription factor. This transcription factor plays an important role in the embryonic development of fibroblasts during the EDFP transition, and therefore can remain expressed in these cells in a quiescent state until adulthood (79–83).

### 4.4 Cellular response to cardiac tissue injury

In response to cardiac tissue injury, TGF-*β* and angiotensin II are released. This activates a signaling cascade in which quiescent resident fibroblasts are activated and express adhesion complexes, including extracellular matrix components. Simultaneously, cell proliferation occurs, mediated by the activation of the p38-MAPK and ERK pathways, promoting and maintaining this proliferation for up to 4 days. Once fibroblasts arrive at the injury site, they express extracellular matrix components through the synthesis of type I and III collagen fibers, fibronectin fibers, and periostin (POSTN) (79–84).

### 4.5 Fibroblast differentiation to myofibroblast

Resident CFs also have the capacity to differentiate into highly specialized contractile myofibroblasts when fibroblasts overproduce fibronectin and collagen, TGF-β, and angiotensin II. Myofibroblasts, unlike fibroblasts, are smooth-muscle-like cells characterized by the presence of contractile actin and myosin filaments, but unlike muscle cells, they also possess specialized adhesion structures. As a cell lineage that differentiates from another cell type after birth, they originate from resident fibroblasts, epithelial cells, endothelial cells, pericytes, and mesenchymal stem cells. The main function of myofibroblasts is to maintain cardiac tissue homeostasis after cardiac injury, as they enable the contraction of adjacent tissue, restore tissue integrity, and facilitate re-epithelialization (79–85).

### 4.6 Myofibroblast differentiation through biomechanical and biochemical pathways

**Figure 3:**
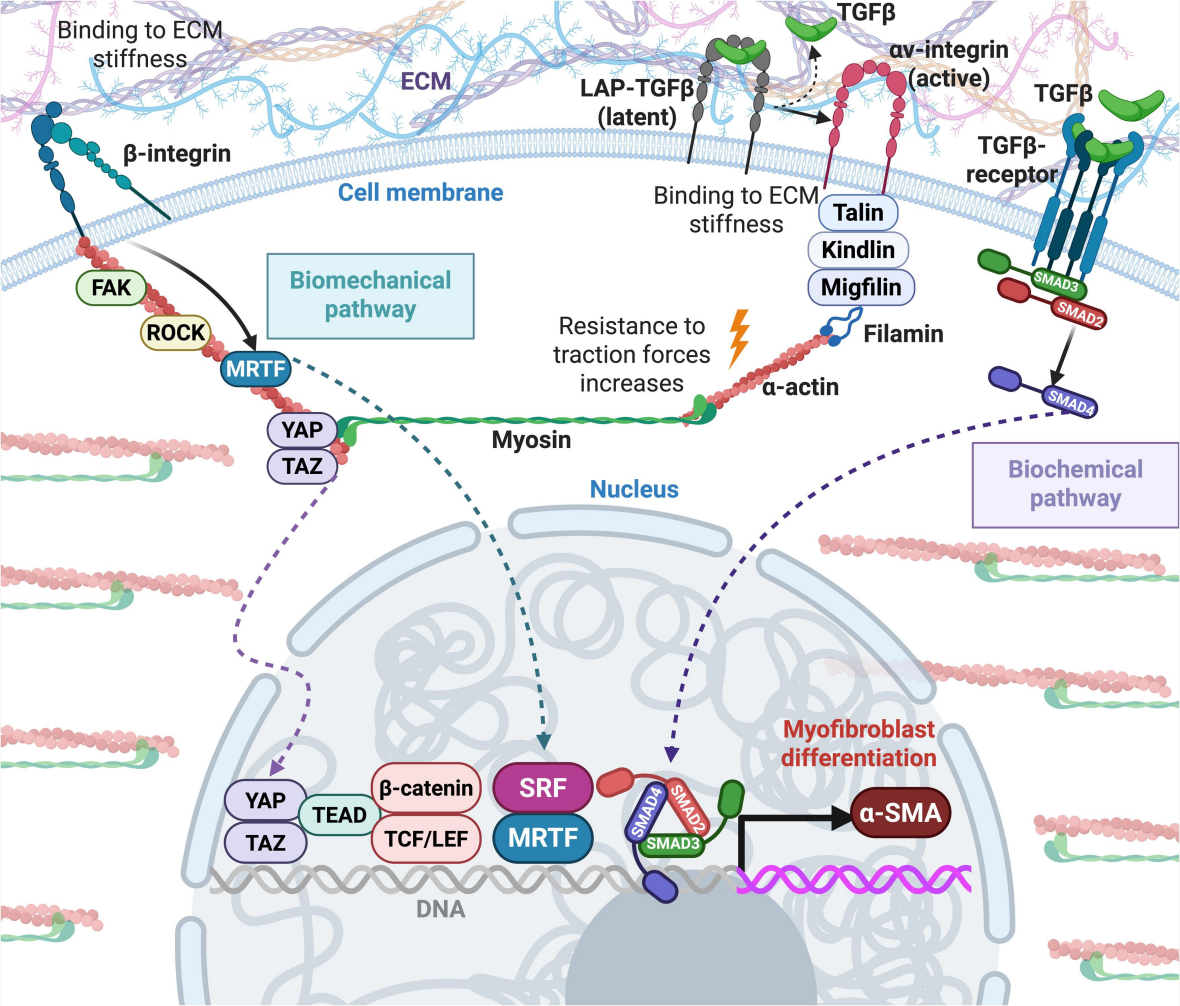
Biomechanical and biochemical pathways for myofibroblast differentiation from fibroblast by inducing alpha-SMA expression, based on (77, 78, 80, 83–85). Original work created at BioRender.com.

Myofibroblast differentiation is mediated by both biomechanical and biochemical pathways (Figure 3). Biomechanical differentiation is driven by the overproduction of extracellular matrix components by fibroblasts, which is simultaneously detected by integrin *β*-1, expressed on the fibroblast plasma membrane, and by an increase in fibroblast tensile strength. This signaling continues intracellularly with the participation of focal adhesion kinase (FAK) and rho-associated protein kinase (ROCK). ROCK enables F-actin stress fibers to allow the nuclear translocation of myocardial-related transcription factor (MRTF), the transcriptional coactivators yes-associated protein (YAP), and the transcriptional coactivator with PDZ-binding motif (TAZ). These factors, together with the serum response factor (SRF), a member of the TEA domain family (TEAD), lymphoid enhancer-binding factor (LEF/TCF), and β-catenin, allow the *de novo* expression of α-SMA, which is encoded by the ACTA2 gene and has its loci on chromosome 10 at 10q22 and 10q24 (75, 77, 79–85).

Simultaneously, biochemical differentiation is also initiated by the rigidity of the extracellular matrix and the traction forces exerted by fibroblasts. For this purpose, fibroblasts possess dimeric integrins called *α*v integrins. As resistance to traction forces increases, the integrins respond to this mechanism, releasing transforming growth factor beta (TGF-β1) from its binding protein stores. Once released, TGF-β1 is activated and binds to its receptors, located on the fibroblast plasma membrane. This binding allows the intracellular activation of SMAD Family Member 3 (SMAD3). Activated SMAD3 binds to SMAD4 to translocate to the nucleus and bind to its binding site on fibrotic gene promoters, activating ACTA2 and expressing α-SMA. Similarly, SMAD3 also promotes FAK activation in the biomechanical pathway, so both mechanisms occur together and are not independent of each other (77, 79, 80, 82–87).

Myofibroblasts, unlike fibroblasts, express the cell markers Fibronectin Extra Domain A (Fibronectin ED-A) and α-SMA, and therefore can also synthesize high amounts of collagen, especially type I collagen. TCF21 also plays a role in cardiac fibrosis: in the event of cardiac injury, the differentiation of fibroblasts into myofibroblasts causes a downregulation of TCF21, which leads to the expression of genes such as ACTA2 and contributes to the proliferation and differentiation of myofibroblasts. This allows TCF21 to be considered a gene repressor, since although its lack of expression in quiescent fibroblasts does not contribute to their differentiation into myofibroblasts, its downregulation during sustained cardiac injury does, driving and perpetuating fibrosis (77, 79–87).

### 4.7 EAT–fibroblast crosstalk

The preceding sections describe the cellular and molecular mechanisms governing fibroblast activation, myofibroblast differentiation, and extracellular matrix deposition in the heart. Because EAT lies in direct anatomical contiguity with the myocardium and lacks an intervening fascial barrier, EAT-derived signals are anatomically positioned to act as paracrine modulators of cardiac fibroblast biology. EAT secretes a complex array of factors with documented or plausible activity on fibroblasts, including adipokines (leptin, resistin, adiponectin), free fatty acids, inflammatory cytokines (TNF-α, IL-6, IL-1β), prostaglandins, and exosomal cargo enriched in microRNAs. Adiponectin, in particular, exerts antifibrotic and anti-inflammatory effects on cardiac fibroblasts in preclinical models, and its reduced output from dysfunctional EAT is consistent with a permissive environment for fibrotic remodeling. Conversely, the leptin-to-adiponectin ratio rises with EAT dysfunction, biasing the local milieu toward fibroblast activation.

The most direct experimental evidence to date comes from Meulendijks et al. (88), who exposed human atrial fibroblasts to the secretome of atrial EAT obtained from patients with and without atrial fibrillation. The EAT secretome from AF patients increased COL1A1 and FN1 expression in atrial fibroblasts approximately fourfold relative to non-AF EAT, with myeloperoxidase identified through proteomic analysis as a major mediator. This finding, together with the recent synthesis of La et al. (89) on epicardial adipocytes in cardiac pathology and healing, supports the concept that EAT-derived signals can produce measurable, biologically meaningful activation of cardiac fibroblasts *ex vivo*.

Whether the magnitude of EAT-mediated fibroblast modulation is sufficient to influence cardiac structure and function *in vivo* remains less firmly established. The strongest evidence is regional and indirect: in patients with atrial fibrillation, fibrotic remodeling is most pronounced in regions of high EAT density, particularly the posterior left atrial wall, and EAT volume correlates with both the extent of atrial fibrosis on imaging and AF recurrence after ablation. In the ventricle, the contribution of EAT to interstitial fibrosis is more difficult to disentangle from confounding effects of obesity, hypertension, and visceral adiposity. We therefore consider the *in vitro* and *ex vivo* evidence for EAT-mediated fibroblast activation to be reasonably robust, the regional clinical evidence in atrial tissue to be supportive, and the *in vivo* causal contribution to ventricular fibrosis to remain partially speculative pending dedicated mechanistic human studies.

## 5 The renin-angiotensin-aldosterone system

### 5.1 Physiology of the renin-angiotensin-aldosterone system

The renin-angiotensin-aldosterone system (RAAS) is a systemic hormonal axis composed of a family of peptides that perform various paracrine and endocrine functions. The RAAS plays a crucial role in regulating blood pressure and maintaining electrolyte balance. Its main target organs are the lungs, kidneys, adrenal glands, cardiovascular system, and brain, although its effects have a systemic impact. Therefore, defects in its function are present in pathologies such as systemic hypertension, heart failure, and nephropathies (90).

### 5.2 Angiotensinogen, renin and angiotensin

The RAAS has angiotensinogen as its precursor, a protein encoded by the AGT gene located on chromosome 1 at 1q42.2. Angiotensinogen is synthesized and released in hepatocytes, where it serves as a substrate for renin, an aspartyl protease encoded by the REN gene located on chromosome 1 at 1q32.1. Renin is found in the form of prorenin in granules within the juxtaglomerular cells of the kidney. Four mechanisms have been described that favor the activation of juxtaglomerular cells, including changes in renal perfusion mediated by baroreceptors; the decrease in sodium and chloride sensed by the macula densa; activation of *β*-1 adrenergic receptors; and negative feedback mediated by other hormones (130). These stimuli trigger an intracellular signaling cascade that cleaves prorenin, releasing its active form, renin, into the bloodstream. Renin has a half-life of 10–15 min and is the rate-limiting enzyme in the RAAS, catalyzing the first step by cleaving angiotensinogen to generate angiotensin I (90–92).

Angiotensin I is an inactive form that circulates in peripheral blood and serves as a precursor to angiotensin II. This conversion requires the angiotensin-converting enzyme (ACE), encoded by the ACE gene located on chromosome 17 at 17q23.3. ACE is produced in the endothelial cells of pulmonary capillaries, peripheral vascular endothelium, and renal tubules. ACE is a transmembrane zinc metalloprotease of the M2 family that cleaves two amino acids from the carboxy-terminal end of angiotensin I, converting it into angiotensin II. ACE has a half-life of 60 s and, once it cleaves angiotensin I into angiotensin II, it is degraded by plasma peptidases into angiotensin III and angiotensin IV (90, 93–97).

Angiotensin II is the primary mediator of the RAAS by playing a significant role in vascular smooth muscle vasoconstriction; inducing the transcription of CYP11B2 for aldosterone synthesis; increasing sodium reabsorption; promoting the release of vasopressin; and increasing the activity of the sympathetic nervous system. There are two types of angiotensin II receptors, both transmembrane and G protein-coupled: AT1R, found mainly in the cardiovascular system, kidneys, adrenal glands, and central nervous system; and AT2R, predominantly found in fetal tissue with expression decreasing in adult tissue (90, 94, 97–100).

### 5.3 Angiotensin G protein-coupled receptors

Both receptors, despite having the same ligand, have opposite physiological effects. AT1R agonism generates an intracellular signaling cascade that regulates vascular smooth muscle contraction by activating myosin light chain kinases, generating vasoconstriction. Furthermore, AT1R agonism results in the activation of intracellular kinases of the MAPK family, which favor the activation of NADPH oxidase and the increase of ROS, generating a persistent pro-inflammatory state while increasing oxidative stress, fibrosis, tissue remodeling and sustained vasoconstriction (90, 101, 102).

The AT2R receptor is considered the main functional antagonist of AT1R, generating vasodilation, inhibition of cell proliferation, and reduction of inflammation and oxidative stress. AT2R agonism leads to the activation of phosphatases, including MKP1, which dephosphorylates MAPK kinases, inhibiting NF-*κ*B. Furthermore, one of the most recognized effects of AT2R agonism is the generation of a vasodilatory effect through the NO-cGMP-bradykinin pathway, in which bradykinin (131) activates endothelial nitric oxide synthase (eNOS), converting L-arginine into nitric oxide (NO). NO activates soluble guanylate cyclase, resulting in arterial vasodilation and reduced blood pressure (103–110, 132).

Another pathway known to have an effect similar to AT2R agonism is the ACE2-mediated pathway. ACE2, encoded by the ACE2 gene on the X chromosome at Xp22.2, is a dipeptidyl carboxypeptidase that cleaves angiotensin I into Ang 1–9 and angiotensin II into Ang 1–7. Ang 1–7 has been observed to become the natural ligand of the MasR receptor, leading to vasodilation and counter-regulating the effects of AT1R (93, 98, 111).

The EAT also contains resident macrophages, which express both AT1R and MasR receptors. In the presence of adipose tissue dysregulation, angiotensin II binding to AT1R on macrophages promotes a pro-inflammatory state and M1 differentiation. The MasR/Ang 1–7 axis has been proposed as a cardioprotective pathway by inhibiting pro-inflammatory interleukins and promoting increased intracellular adiponectin, reducing lipotoxicity and insulin resistance (93, 98, 111).

### 5.4 Aldosterone

One of the functions of angiotensin II is to induce the transcription of CYP11B2, located on chromosome 8 at 8q24.3, encoding aldosterone synthase in the zona glomerulosa of the adrenal cortex for aldosterone synthesis. Aldosterone is a mineralocorticoid derived from cholesterol with a half-life of 20 min. Its main functions are to maintain fluid and electrolyte balance through epithelial sodium channels (ENaCs) and the Na+/K+ ATPase pump. Mineralocorticoid receptors are also found in brain areas responsible for central regulation of fluid intake and sympathetic nervous system activity (90, 112–115).

### 5.5 EAT–RAAS bidirectional interaction

The preceding sections describe the RAAS as a systemic hormonal axis with broad cardiovascular effects. EAT, however, expresses the principal RAAS components locally, including angiotensinogen, ACE, ACE2, AT1R, AT2R, and the Mas receptor, and is therefore not merely a passive target of circulating RAAS activity but a tissue capable of intrinsic RAAS signaling. Conversely, systemic RAAS activation appears to feed back on EAT itself, with effects on EAT mass, inflammatory tone, and secretory profile. The interaction is therefore bidirectional: EAT modulates RAAS activity in its anatomical vicinity, and systemic RAAS activity modulates the biology of EAT. This bidirectionality has important implications for understanding how EAT may contribute to cardiac pathology.

The most directly informative experimental evidence comes from the work of Patel et al. (98), in which loss of ACE2 in a murine model produced a heart failure with preserved ejection fraction (HFpEF) phenotype mediated, at least in part, through EAT inflammation. In the same model, administration of Ang(1–7) attenuated cardiac dysfunction predominantly via effects on EAT adiponectin expression and the resolution of EAT inflammation, rather than through direct myocardial effects alone. This finding provides direct experimental support for the concept that perturbation of the ACE2/Ang(1–7)/Mas axis at the level of EAT translates into measurable cardiac dysfunction, and that restoration of this axis can attenuate that dysfunction through EAT-mediated mechanisms. The recent synthesis by Qi et al. (116) integrates EAT, RAAS, and cardiac dysfunction in HFpEF within a unified molecular framework, situating these findings within the broader pathophysiology of the syndrome.

Taken together with the considerations raised in the Introduction regarding the partial blood-pressure dependence of RAAS-mediated cardiac effects, this bidirectional EAT–RAAS interaction suggests a model in which local EAT-derived RAAS components contribute to a tonic, low-grade pro-inflammatory and pro-fibrotic milieu in the immediate vicinity of the myocardium, while systemic RAAS activation accompanied by hemodynamic change produces more pronounced, pressure-dependent fibrotic remodeling. The relative contributions of these two arms remain incompletely resolved and represent an important area for future mechanistic human studies.

## 6 Arrhythmias

### 6.1 Introduction to arrhythmias

An arrhythmia is an abnormal heart rhythm. The cardiac conduction system (CCS) operates through a pacemaker composed of specialized cardiomyocytes that generate electrical impulses—the sinoatrial (SA) node. Once depolarization begins, the impulse travels through internodal tracts to the atrioventricular (AV) node, continues through the bundle of His, divides into its right and left branches, and finally reaches the Purkinje fibers for effective ventricular conduction (117).

### 6.2 Atrial fibrillation

Atrial fibrillation (AF) is the most common arrhythmia and the most frequent tachyarrhythmia, associated with high mortality due to the risk of embolism and stroke. AF is caused by the absence of effective atrial contraction, resulting in multiple disorganized impulses occurring in different areas within the atrium. On electrocardiogram, this manifests as irregular ventricular activity with f waves (117, 118).

### 6.3 The role of EAT in abnormal electrical conduction activity

A pathological increase in EAT leads to a pro-inflammatory state characterized by the release of pro-inflammatory cytokines, which promote cardiac remodeling and abnormal transmission of the cardiac impulse. A recent study by Fan et al. used an *in vitro* tridirectional culture model to reveal an adipocyte-neuronal-proarrhythmic axis. In this model, leptin secreted by epicardial adipocytes activates sympathetic neurons, stimulating the release of neuropeptide Y (NPY), which increases the activity of the Na+/Ca2+ exchanger (NCX) and calcium-calmodulin-dependent protein kinase II (CaMKII), raising the intracellular calcium load and potentially leading to arrhythmia (119).

The EAT surrounds cardiac ganglion plexuses and serves as a storage site for catecholamines such as norepinephrine. An increase in EAT leads to pathological norepinephrine release, increasing the sympathetic response and generating arrhythmias (119, 120).

Dysfunctional EAT secretes pro-inflammatory and pro-fibrotic cytokines, including IL-6 and TNF-α. IL-6 induces JAK/STAT3 signaling, which reduces connexin expression, promoting atrial electrical remodeling. TNF-α activates fibroblasts via TGF-β and increases MMP2, promoting collagen deposition. EAT also increases MCP-1 secretion, recruiting macrophages that stimulate myofibroblast differentiation, leading to extracellular matrix thickening and electrical instability (119–121).

Altered EAT metabolism increases ROS production. Epicardial leptin exacerbates mitochondrial oxidative stress via the PKC/NADPH oxidase pathway. Elevated ROS promotes CaMKII oxidation, increasing spontaneous calcium leakage from the sarcoplasmic reticulum through ryanodine receptors, leading to arrhythmogenic delayed potentials. At the structural level, fat infiltration in the myocardium generates histological discontinuities, forming adipose islands between myocyte fascicles that alter tissue anisotropy and favor re-entry circuits (64, 119, 121).

At the electrical level, soluble factors from EAT directly modulate cardiac ion channels. An *in vitro* study demonstrated that the epicardial adipose tissue secretome drastically slows conduction velocity, corresponding to lower excitability due to partial sodium channel inactivation and a raised activation threshold, increasing the probability of reentrant arrhythmias (64, 119, 122, 123).

## 7 Clinical implications of EAT

### 7.1 EAT measurement

Several methods for measuring EAT have been described, including echocardiography, MRI, and CT (Table 2). Echocardiographic measurement has shown advantages as a practical and inexpensive approach. Iacobellis et al. were the first to propose an echocardiographic approach using standard parasternal long-axis and short-axis views (124). This approach allows visualization of EAT thickness, measured perpendicular to the free wall of the right ventricle at end-systole over three cardiac cycles. However, one limitation of echocardiography is that it provides a linear measurement at a single location, making it difficult to distinguish irregularities in EAT distribution. Three-dimensional echocardiography has been proposed to overcome these limitations (125).

**Table 2:** Imaging modalities for EAT measurement, based on (11, 21).

**Imaging Modality**	**Advantages**	**Disadvantages**
Echocardiography	Allows measurement of EAT thickness. Low-cost, non-invasive method.	Interoperator variability. Dependent on measurement location. Does not allow measurement of EAT volume.
CT	Allows measurement of EAT thickness and volume. Suitable for regional EAT measurement (pericoronary, peri-atrial).	High cost. Limited availability. Radiation exposure.
MRI	Allows measurement of EAT thickness and volume. Suitable for regional EAT measurement. No radiation exposure.	High cost. Limited availability.

Morales-Portano et al. conducted a study in Mexico City comparing EAT thickness with other markers in predicting MACEs. The results demonstrated that EAT thickness was four times more predictive of MACEs than BMI, LVEF, and coronary artery complexity. However, variability of results depending on measurement site compounded the lack of current guidelines or consensus on clinical use of EAT measurement (11).

EAT volume, measured using CT and MRI, has proven to be an effective biomarker related to cardiovascular disease risk. Both MRI and CT offer advantages over echocardiography as three-dimensional approaches. MRI is currently considered the gold standard for evaluating adipose tissue, as it allows reproducible assessment of volumes, mass, and cardiac function from early stages without radiation exposure (126, 127).

### 7.2 Correlations of EAT, obesity and metabolic syndrome

Obesity and metabolic syndrome induce profound systemic hemodynamic, neurohormonal, and metabolic alterations that promote adverse cardiac remodeling and ventricular dysfunction, even in the absence of overt coronary artery disease or systemic hypertension. Within this framework, EAT should be regarded not merely as a passive marker of cardiometabolic risk, but as an active participant in the pathophysiology of metabolic syndrome-associated cardiac remodeling, coronary atherosclerosis, and myocardial dysfunction (7).

#### 7.2.1 Adipocyte hypertrophy and inflammatory phenotype

Excess caloric intake and systemic insulin resistance promote adipocyte hypertrophy within epicardial fat, leading to expansion of triglyceride deposits and a shift in metabolic behavior. Hypertrophied epicardial adipocytes demonstrate increased lipolytic activity and enhanced release of free fatty acids, which may diffuse directly into adjacent myocardial tissue. Adipocyte enlargement is associated with activation of inflammatory signaling pathways, specifically through increased expression of TNF-α and MCP-1 (15). The absence of a fibrous fascial barrier between epicardial fat and the myocardium increases the impact of adipocyte hypertrophy, allowing inflammatory mediators and lipid metabolites to readily access neighboring cardiac structures.

#### 7.2.2 Mitochondrial and thermogenic remodeling

Under physiological conditions, EAT exhibits a beige-like phenotype characterized by elevated mitochondrial density and expression of thermogenic genes such as UCP-1, PRDM16, and PGC-1*α*. In obesity, metabolic syndrome, and CAD, EAT undergoes molecular reprogramming characterized by reduced expression of thermogenic regulators and increased expression of white adipocyte markers. This process, described as “beige-to-white transition” or “whitening,” is accompanied by decreased uncoupled respiration, enhanced triglyceride storage, and acquisition of a pro-inflammatory secretory profile. Qualitative alterations in mitochondrial activity and thermogenic capacity may be more relevant than EAT volume alone in determining cardiovascular risk (7, 73).

#### 7.2.3 Immune cell infiltration and chronic low-grade inflammation

As EAT hypertrophy progresses, it develops a pronounced inflammatory microenvironment characterized by immune cell infiltration and sustained low-grade inflammation. MCP-1 plays a central role by promoting monocyte migration into the stromal-vascular matrix of epicardial fat. These infiltrating macrophages secrete pro-inflammatory cytokines, including TNF-α, IL-6, and IL-1β. The persistence of this inflammatory environment creates a self-amplifying cycle in which cytokine release further impairs insulin signaling, increasing lipolysis and perpetuating FFA release (15, 21).

#### 7.2.4 Dysregulated adipokine secretion

In MetS, EAT exhibits profound dysregulation of adipokine secretion, with reduced adiponectin expression and increased expression of pro-inflammatory adipokines including leptin, resistin, PAI-1, and angiotensinogen. These mediators contribute to endothelial dysfunction, increase oxidative stress, and impair insulin signaling (15, 16, 21).

#### 7.2.5 Paracrine and vasocrine signaling between EAT and the heart

A distinctive feature of EAT is its direct anatomical contiguity with the myocardium and coronary arteries without an intervening fascial barrier. Paracrine signaling allows pro-inflammatory cytokines, adipokines, and FFAs to diffuse through the interstitial space into the underlying myocardium and coronary arteries. Vasocrine signaling provides an additional route whereby adipokines access the coronary vasa vasorum and travel to deeper vascular layers. Both mechanisms operate simultaneously in MetS, contributing to adverse ventricular remodeling, myocardial inflammation, and coronary atherosclerosis (15, 74).

#### 7.2.6 Free fatty acid flux and cardiac lipotoxicity

In metabolic syndrome, adipocyte hypertrophy, insulin resistance, and enhanced lipolytic activity lead to excessive FFA release. Sustained exposure of cardiomyocytes to elevated FFA concentrations promotes myocardial lipid accumulation and formation of toxic lipid intermediates such as ceramides, disrupting intracellular signaling pathways and leading to cardiomyocyte dysfunction and apoptosis. This lipotoxic environment contributes to myocardial steatosis, impaired diastolic function, ventricular remodeling, and increased susceptibility to heart failure (15, 123, 128, 129).

#### 7.2.7 Insulin resistance and local metabolic dysregulation

Epicardial fat thickness and volume correlate more closely with insulin resistance and visceral adiposity than with overall BMI. Within EAT, impaired insulin signaling promotes unchecked lipolysis, increased FFA release, and further dysregulation of adipokine secretion. This localized insulin-resistant state facilitates a vicious cycle in which inflammation, lipotoxicity, and metabolic dysregulation perpetuate one another, linking systemic metabolic abnormalities to local cardiac inflammation, remodeling, and dysfunction (7, 15, 21).

## 8 Therapeutic implications and future perspectives

The growing characterization of EAT as a metabolically and endocrinologically active visceral fat depot has prompted exploration of EAT as a therapeutic target. GLP-1 receptor agonists, such as liraglutide and semaglutide, have been reported to reduce EAT volume in observational and small interventional studies. Proposed mechanisms include effects on adipocyte metabolism and, in preclinical models, browning of white adipose tissue through hypothalamic AMPK activation. It should be emphasized, however, that whether EAT volume reduction mediates cardiovascular benefit independently of weight loss, glycemic control, and broader effects on visceral adiposity has not been definitively established. Strategies targeting mitochondrial restoration and “re-browning” of EAT remain at the preclinical and conceptual stage and should be considered exploratory therapeutic directions rather than established interventions.

The identification of EAT as both a biomarker and a mediator of cardiovascular disease positions it as a cornerstone for future precision medicine approaches. Standardization of EAT measurement parameters across imaging modalities, along with the development of EAT-specific therapeutic targets, will be essential for translating current research findings into clinical practice. Future studies should focus on prospective clinical trials evaluating the impact of EAT-directed therapies on hard cardiovascular endpoints.

## 9 Limitations and knowledge gaps

Despite the substantial growth in EAT research over the past two decades, several important limitations and unresolved questions warrant explicit acknowledgment. First, EAT measurement remains methodologically heterogeneous. Echocardiographic thickness, cardiac CT volumetric quantification, and cardiac MRI measurements each capture different aspects of EAT and yield non-interchangeable values. Standardized cut-off thresholds for risk stratification have not been established across imaging modalities, and inter-observer variability remains a practical limitation, particularly for echocardiographic measurement. Until consensus thresholds and standardized acquisition and analysis protocols are established, comparison of findings across studies and translation to routine clinical practice will remain constrained.

Second, confounding between EAT and general visceral adiposity remains a persistent methodological challenge. EAT shares embryological origin, secretory profile, and many systemic correlates with visceral abdominal fat, and disentangling the independent contribution of EAT—as opposed to visceral adiposity as a whole—to cardiovascular outcomes requires careful statistical adjustment that is not consistently performed across the available literature. The degree to which EAT-specific signals add prognostic information beyond established markers of visceral adiposity therefore remains incompletely resolved.

Third, the available evidence base is dominated by observational data. Cross-sectional and prospective cohort studies have established robust associations between EAT volume or thickness and a range of cardiovascular outcomes, but few prospective interventional trials have been specifically designed to test whether modification of EAT—rather than concurrent reductions in weight, glycemic burden, or visceral adiposity—mediates cardiovascular benefit. The recent interest in GLP-1 receptor agonists has generated reports of EAT volume reduction, but definitive evidence that this effect contributes independently to cardiovascular benefit beyond established mechanisms of weight loss and glycemic control is currently lacking.

Fourth, much of the mechanistic literature on EAT biology derives from rodent models, with attendant limitations regarding species differences. EAT in rodents differs from human EAT in volume, anatomical distribution, and to some extent in secretory profile, and the translational generalizability of rodent findings should be interpreted with appropriate caution. Where possible, mechanistic claims in this review are anchored to human *ex vivo* or *in vivo* data; where rodent or *in vitro* evidence is the primary basis, this is explicitly noted.

Finally, the present review is a narrative synthesis rather than a systematic one. While search strategy and prioritization criteria have been made explicit (Section 2, Methods), formal risk-of-bias assessment of included studies and quantitative synthesis were not performed. The potential for selection bias inherent to this format is acknowledged.

### 9.1 Priorities for future research

Several priorities for future research follow directly from the limitations identified above. First, the field requires consensus standardization of EAT quantification, including harmonized acquisition protocols, analysis pipelines, and reference thresholds across echocardiography, cardiac CT, and cardiac MRI. Second, prospective outcomes studies specifically designed to validate EAT-based risk stratification—ideally evaluating incremental value over established cardiovascular risk scores—are needed before EAT measurement can be confidently incorporated into clinical guidelines. Third, dedicated mechanistic human studies of EAT–myocardium crosstalk, including paired analysis of EAT secretome, cardiac fibroblast activation markers, and structural cardiac imaging, would substantially advance the field beyond its present reliance on rodent and *ex vivo* models. Fourth, the development of EAT-directed therapeutic strategies—whether through repurposing of existing agents with documented EAT effects (such as GLP-1 receptor agonists, GLP-1/GIP co-agonists, SGLT2 inhibitors, and mineralocorticoid receptor antagonists) or through novel approaches targeting EAT-specific biology—should be evaluated in trials designed with EAT-mediated mechanisms as a prespecified outcome. Addressing these priorities is essential for transforming the current understanding of EAT into clinically actionable cardiovascular care.

## 10 Conclusions

Epicardial adipose tissue is increasingly recognized as a metabolically and endocrinologically active visceral fat depot with relevance to cardiovascular health and disease. Its anatomical position in direct contact with the myocardium, shared vascularization through the coronary circulation, and capacity for paracrine and vasocrine signaling distinguish it from other adipose tissue depots and are consistent with a role as a regional mediator of cardiac pathophysiology. Under physiological conditions, EAT contributes to local metabolic support, mechanical protection, and thermogenic regulation of the heart. In the context of obesity, metabolic syndrome, and coronary artery disease, EAT undergoes structural and functional remodeling characterized by adipocyte hypertrophy, immune cell infiltration, and a shift from a beige-like phenotype toward a pro-inflammatory white phenotype.

The mechanisms by which dysfunctional EAT contributes to cardiac fibrosis, arrhythmogenesis, and coronary atherosclerosis are becoming increasingly well-characterized. The RAAS, particularly the AT1R-mediated pro-inflammatory pathway, plays a central role in EAT-related cardiac pathology, while the ACE2/Ang 1–7/MasR counter-regulator*y* axis offers potential therapeutic targets. Furthermore, the identification of EAT thickness and volume as independent predictors of adverse cardiovascular events underscores its value as a clinical biomarker.

As measurement techniques become standardized and EAT-specific therapies advance, epicardial adipose tissue is poised to become a central target in the prevention and treatment of cardiovascular disease.
